# Lack of netrin-4 modulates pathologic neovascularization in the eye

**DOI:** 10.1038/srep18828

**Published:** 2016-01-06

**Authors:** Norbert Kociok, Sergio Crespo-Garcia, Yong Liang, Sabrina V. Klein, Christina Nürnberg, Nadine Reichhart, Sergej Skosyrski, Eva Moritz, Anna-Karina Maier, William J. Brunken, Olaf Strauß, Manuel Koch, Antonia M. Joussen

**Affiliations:** 1 Department of Ophthalmology, Charité Universitätsmedizin, Berlin, Germany; 2 Department of Ophthalmology, Peking University, People’s Hospital, Beijing, China; 3 Institute for Dental Research and Oral Musculoskeletal Biology and Center for Biochemistry, Medical Faculty, University of Cologne, Cologne, Germany; 4 Department of Ophthalmology, Upstate Medical University, Syracuse, New York, USA

## Abstract

Netrins are a family of matrix-binding proteins that function as guidance signals. Netrin-4 displays pathologic roles in tumorigenesis and neovascularization. To answer the question whether netrin-4 acts either pro- or anti-angiogenic, angiogenesis in the retina was assessed in *Ntn-4*^−/−^ mice with oxygen-induced retinopathy (OIR) and laser-induced choroidal neovascularization (CNV), mimicking hypoxia-mediated neovascularization and inflammatory mediated angiogenesis. The basement membrane protein netrin-4 was found to be localised to mature retinal blood vessels. Netrin-4, but not netrin-1 mRNA expression, increased in response to relative hypoxia and recovered to normal levels at the end of blood vessel formation. No changes in the retina were found in normoxic *Ntn-4*^−/−^ mice. In OIR, *Ntn-4*^−/−^ mice initially displayed larger avascular areas which recovered faster to revascularization. Ganzfeld electroretinography showed faster recovery of retinal function in *Ntn-4*^−/−^ mice. Expression of netrin receptors, Unc5H2 (Unc-5 homolog B, *C. elegans*) and DCC (deleted in colorectal carcinoma), was found in Müller cells and astrocytes. Laser-induced neovascularization in *Nnt-4*^−/−^ mice did not differ to that in the controls. Our results indicate a role for netrin-4 as an angiogenesis modulating factor in O_2_-dependent vascular homeostasis while being less important during normal retinal developmental angiogenesis or during inflammatory neovascularization.

The retina is a complex system composed of highly specialised primary sensory cells (photoreceptors) and neuronal cells as well as blood vessels. Previous research on vascular disease in the retina has focused mainly on the role of vascular alterations in onset and development of disease. However, little attention has been given to the contribution of neurovascular interactions and coupling^1^.

Throughout the body, blood vessels and neuronal cells build complex systems that are anatomically and patho-physiologically intertwined; in the periphery of the body larger nerves are dependent on vascularization to ensure nutrition and oxygen supply, whereas arteries depend on innervation^2^. This mutually beneficial interaction has been shown to be necessary for development of neuronal tissues, with axonal path finding molecules such as netrins controlling both vessel morphogenesis and angiogenesis^3^. Netrins are a family of laminin-related extracellular matrix (ECM) molecules with homology to the short arm of laminin molecules. They function as long range guidance signals, acting in early embryogenesis regulating the migration of neurons and the axon growth cones^4^,^5^ and have been shown to induce neurite growth^6^. In mammals, five members of the netrin family have been identified: netrin-1^4^, netrin-3^7^,^8^, netrin-4^6^,^9^ netrin-G1 and netrin-G2^10^,^11^. Netrin-1, -3 and -4, are secreted into the ECM, while netrin-G1 and -G2 are bound to the cell surface via a glycosylphosphatidylinositol link.

The functional role of the newest member, netrin-4, is not well understood. Netrin-4, a 628 amino acid protein^6^, is similar in size and secondary structure to that of the other netrins; however, in terms of its primary structure, netrin-4 appears to be a distant relative. The amino acids of its globular domain are more closely related to that of the laminin β-chains^9^. *In vitro*, netrin-4 promotes neurite growth from E14 rat olfactory bulb explants, suggesting it may be important as a haptotactic factor promoting axonal elongation or as a substrate for neuronal migration^6^. In addition, netrin-4 is located in the basement membranes of blood vessels and is therefore thought to play a key role in angiogenesis^6^,^9^. Netrin-4 is highly expressed in the retina. This expression occurs in all ocular tissues as a component of the basement membranes^12^.

In line with other members of the netrin family, which have been implicated to play a role in various diseases, netrin-4 seems to be important mainly for tumours and pathologic neovascular tissues: it serves as a prognostic marker in tumours and is reported to delay/decrease their development, seemingly through restricting blood vessel growth^13^. Despite accumulating evidence suggesting a role for netrin-4 in angiogenesis^13^,^14^,^15^, its exact function remains controversial; for example, netrin-4 morpholino knock-down in embryonic zebrafish results in altered blood vessel formation^16^, whereas Park *et al.*^17^ have observed only a weak expression of zebrafish netrin-4 during embryonic development. Other observations showed an altered netrin-4 expression related to cancer development and pathological vessel growth: in an *in vivo* model of diabetic mouse limb ischemia, angiogenesis was promoted following cerebral ischemia^18^. In the retina, netrin-4 expression is driven by hypoxia, further highlighting its pathologic function^19^. In addition, netrin-4 is up-regulated in VEGF-stimulated endothelial cells, reducing pathological angiogenesis *in vitro* and in laser-induced choroidal neovascularization^14^.

Here we studied the specific angiogenic function of netrin-4. We employed a netrin-4 deficient mouse model, with clinical relevance for pathologic neovascularization in the retina and choroid, to test the hypothesis that netrin-4 plays an important regulatory role in pathologic angiogenesis while having a less important role in developmental angiogenesis.

## Results

### Differential expression of netrin-4 and netrin receptors in the wild type mouse retina during postnatal development

**Figure 1:**
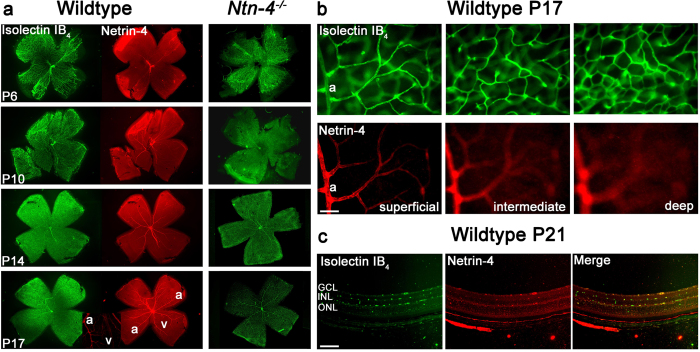
Netrin-4 expression in WT mice and phenotype of *Ntn-4*^−/−^ retina. (**a**) Vessel development and netrin-4 expression in WT and *Ntn-4*^−/−^ mice. Vessels were visualised with Isolectin-IB_4_ staining in both genotypes (left and right panel), netrin-4 was stained only in WT mice (middle panel) from P6 to P17. No obvious expression of netrin-4 is detected at P6 in WT mice. Only the persistent hyaloid artery stained positive for netrin-4. While the retina is almost fully vascularized at P10 and P14, the netrin-4 staining is still limited to the large vessels. Vascularization of the *Ntn-4*^−/−^ mice does not differ from WT. At P17 there is netrin-4 expression throughout the retinal vasculature up to the capillary level. The insert shows a preferential netrin-4 staining of arterioles. a = arteries, v = veins (**b**) Co-staining against netrin-4 and Isolectin-IB_4_ in flat-mounts of WT retinas at P17. Expression of netrin-4 was found in the main branching retinal vessels and the superficial capillaries (left panel) but not in the intermediate and the deep capillaries (right panel). Scale bar indicates 100 μm. (**c**) Co-staining against netrin-4 and Isolectin-B4 on sections from WT mice retinas at P21. Netrin-4 expression is present at the level of Bruch´s membrane, but now also in the larger intermediate and deep retinal capillaries. Scale bar indicates 50 μm.

Due to accumulating evidence that netrin-4 is involved in embryonic development, we investigated the hypothesis that netrin-4 is a regulator of angiogenesis, and examined retinal netrin-4 expression during normal postnatal development in mice using immunohistochemistry. While netrin-4 deposition in the basement membrane of the retina has been examined in a prior study^12^; we specifically studied netrin-4 expression with reference to the retinal vascular development which develops entirely postnatally^20^,^21^. At P6, netrin-4 expression was not detected in retina sagittal sections and flat mounts; however, staining of the hyaloid artery and its branches was observed in retinal flat mounts (Fig. 1a). Sparse staining for netrin-4 in the larger vessels of the retinal vasculature was found beginning at P10 (Fig. 1a). There was a notable regression of the hyaloid artery from P10 to P17. While the peripheral retina was already fully vascularized at P10, there was netrin-4 staining initially only in large vessels. At P17, netrin-4 staining was visible throughout the retinal vasculature up to the capillary level; staining in superficial layers of the arterial side in the vascular tree was prominent, but absent from deeper layers (Fig. 1b). At P21 Netrin-4 expression was also present in the deep capillaries and the ocular basement membranes, including Bruch´s membrane (Fig. 1c). These observations indicate netrin-4 expression is associated with the maturation of postnatal blood vessels and is preferentially concentrated in the arterial and micro vessel basement membranes during vessel development. In the adult retina, netrin-4 expression was more evident on arterioles than on veins (Fig. 1a, insert and Suppl. Fig. 1). Identification of retinal arteries and veins was done morphologically as described^22^.

### Effect of netrin-4 on the postnatal development of the retinal vasculature in *Ntn-4* ^−/−^mice

The differential netrin-4 expression might indicate a minor role of netrin-4 in the early formation of blood vessels which is in accordance with Li *et al.*^12^, who suggested that netrin-4 plays a very minor role in normal eye development. Thus, we investigated the effect of netrin-4 deficiency on retinal vasculature and studied the formation of retinal blood vessels in netrin-4 deficient mice (*Ntn-4*^−/−^) at various time points of postnatal development (P6-P21), again using immunostaining (Fig. 1a, right panel). Lack of netrin-4 staining of *Ntn-4*^−/−^ retinal flat mounts at P6 and in adult mice confirmed the absence of netrin-4 in this mouse line (Suppl. Fig. 2). The retinal vasculature remained largely unaltered exhibiting, as in WT mice, incomplete vascularization at P6 (Suppl. Fig. 3) and fully vascularized retinas from P10 on. Branching of the vessels as well as capillary formation appeared as in the wild type (Fig. 1a, right panel).

In both mice genotypes, co-staining of neovascular tufts (Isolectin-IB_4_) and pericytes (NG2) revealed a normal pericyte distribution in the retinal vessels during physiological conditions. We detected a pronounced pericyte staining in the neovascular tufts (Suppl. Fig. 4). Significant differences between the two genotypes were not detected. Moreover, partial co-staining of isolectin IB_4_ and laminin γ3 in the WT also showed only limited association between laminin γ3 and the pathological vessels (Suppl. Fig. 5).

Thus we confirmed that netrin-4 is not required for physiological development of retinal blood vessels^12^.

### Faster recovery from OIR vascular damage in *Ntn4* ^−/−^ mice

**Figure 2:**
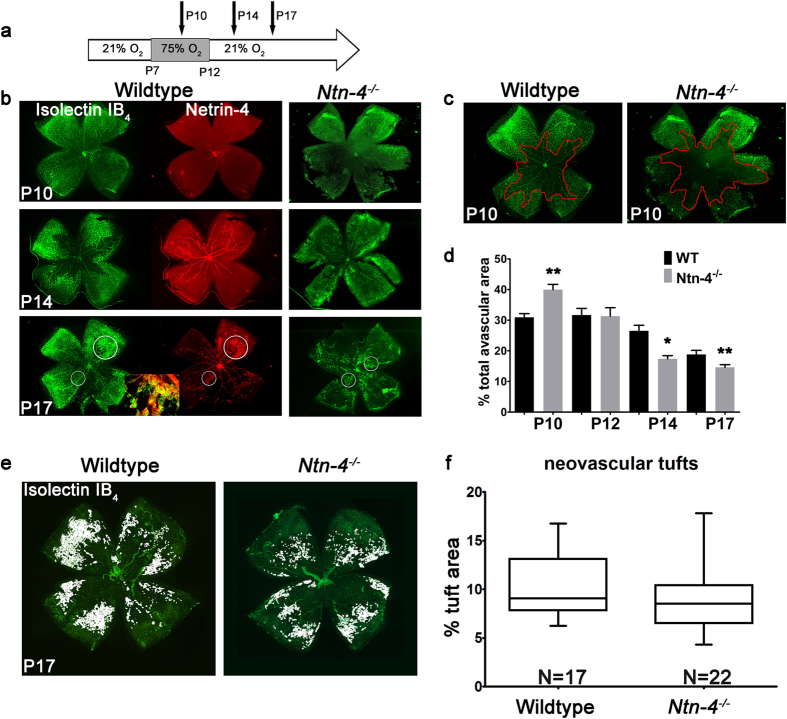
Faster morphological recovery from OIR vascular damage in *Ntn-4*^−/−^ mice. Netrin-4 expression and development of retinal blood vessels in *Ntn-4*^−/−^ and their respective WT mice after oxygen challenge. (**a**) Experimental paradigm: oxygen exposure lasted from P7 to P12. (**b**) Retina flat-mounts at P10, P14 and P17.Co-staining against netrin-4 (red) and Isolectin- IB_4_ (green) demonstrate netrin-4 expression on the large retinal vessels at P10, P14 and P17 with prominent staining in the areas of pathological retinal neovascularization (Insert: composite of netrin-4 staining (red) and Isolectin- IB_4_ staining (green)). *Ntn-4*^−/−^ mice also demonstrate a prominent neovascular response on P17 (right panel). (**c**) Example of avascular area quantification in *Ntn-4*^−/−^ and their respective WT controls at P10. Red line outlines the capillary edges between vascular and non-vascular areas to define the area used for quantification. (**d**) Quantification of the avascular areas of *Ntn-4*^−/−^ and their respective WT mice. Avascular area is given as percentage of total retinal area. At P10 the avascular area was significantly larger in *Ntn-4*^−/−^ mice but decreased faster in *Ntn-4*^−/−^ mice than in the WT, beginning at P14 (n = 4). *p < 0.05; **p < 0.01. (**e**) Isolectin-IB4 stained retinal flat mounts at P17 of WT (left) and *Ntn 4*^−/−^ mice (right). Neovascular tufts are highlighted in white. (**f**) Quantification of neovascular tufts. The neovascular tufts showed a tendency to be more reduced in *Ntn 4*^−/−^ mice compared to the WT. N = number of flat-mounts.

The developmental sequence of netrin-4 expression and its restricted spatial expression suggests that it might play a role in the stabilisation of mature blood vessels and perhaps vascular remodelling. Therefore, we undertook an analysis of the role of netrin-4 in pathological blood vessel formation using an oxygen induced retinopathy (OIR) mouse model (Fig. 2a). Retina flat-mounts at P10, P14 and P17 demonstrated a similar time course of netrin-4 expression as at normoxia (Fig. 1). In addition, immunostaining revealed netrin-4 expression in the large tortuous retinal vessels on P14 and P17 with prominent staining of the areas of retinal neovascularization (Fig. 2b). Especially the neovascular tufts exhibit a prominent co-staining of netrin-4 and Isolectin-IB_4_ (Fig. 2b, insert) at a time point when at normoxia only the superficial capillaries are stained. Compared to wild-type mice, *Ntn4*^−/−^ mice exhibited similar neovascular tufts at P17 (Fig. 2b, lower row). By P28, the pathological tufts and vascularization vanished both in WT as well as in *Ntn-4*^−/−^mice.

As an indicator of blood vessel development, the size of the avascular zone was measured in retina flat-mount preparations. The avascular zone in P10 WT was significantly smaller compared to that in *Ntn-4*^−/−^ mice (30.9% ± 1.2%; N = 4 versus 39.9% ± 1.7%; N = 5; p = 0.005) (Fig. 2c,d). By P12, the avascular zone was equally large in the retina of both mouse strains (31.7% ± 2.2% (N = 5) and 31.3% ± 2.7% (N = 3); p = 0.926), but at later time points was significantly reduced in size in *Ntn-4*^−/−^ mice: At P14 the mean avascular zone in the WT was 26.5% ± 1.8% (N = 6) compared to 17.4% ± 1% (N = 3) in *Ntn-4*^−/−^ mice (p = 0.013). At P17 the mean avascular zone in WT animals measured 18.8% ± 1.3% (N = 17) of total retina surface area compared to 14.6% ± 0.8% (N = 22) in *Ntn-4*^−/−^ mice (p = 0.009) (Fig. 2d). Thus in the absence of netrin-4, closure of the avascular area by new vessels is accelerated.

Since in the *Ntn-4*^−/−^ mice this occurred faster, we also analysed the neovascular tufts in more detail. Neovascular retinal tufts were identified as hyper fluorescent irregular formed vessels at P17 (Fig. 2e) as indicated^23^,^24^. Some neovascular vessels in *Ntn-4*^−/−^ mice appear as large branched pathological vessels compared to the more circumscribed neovascular tufts in WT and showed a tendency to be more reduced in *Ntn-4*^−/−^ mice compared to WT; however, this didn’t turn significant (Fig. 2f). However, an analysis of tufts in H/E-stained sections showed a significant reduction in the amount of epiretinal nuclei in *Ntn-4*^−/−^ mice compared to the WT (Suppl. Fig. 6).

**Figure 3:**
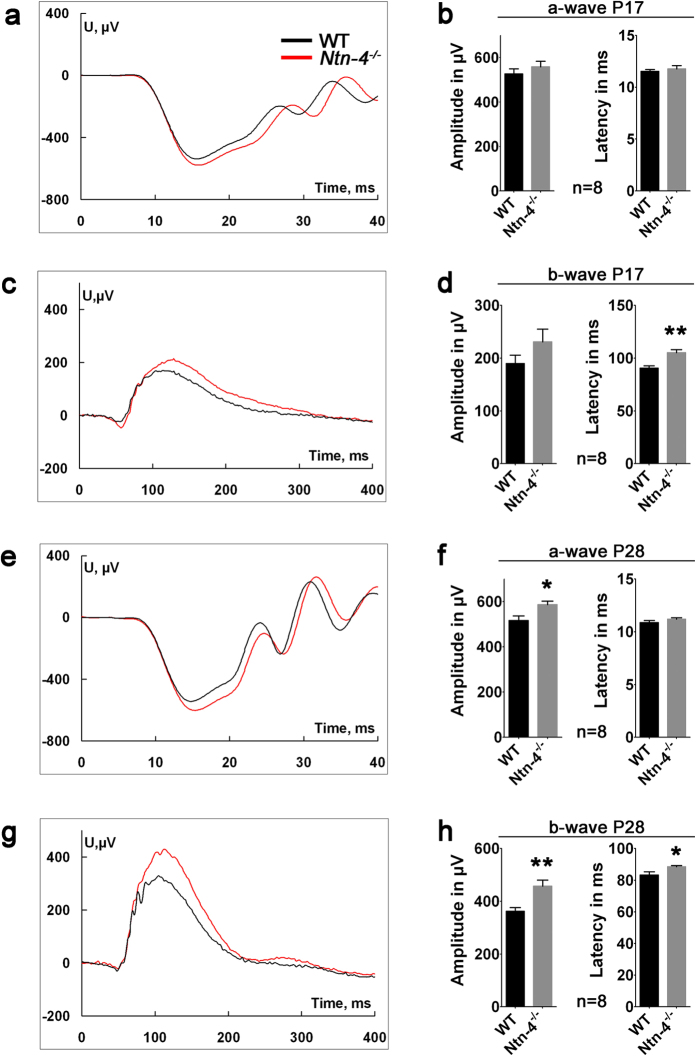
Faster physiological recovery from OIR vascular damage in *Ntn-4*^−/−^ mice. Ganzfeld-ERGs at P17 and P28 in oxygen-treated mice (**a**) Scotopic a-waves in response to a single flash of 10 cds/m^2^ in *Ntn-4*^−/−^ (red) and WT (black) at P17 (**b**) Comparison of a-wave amplitude at 10mcds/m^2^ (left panel) and latency (right panel). (**c**) Scotopic b-waves in response to single flash 10 mcds/m^2^ in *Ntn-4*^−/−^ (red) and WT (black) at P17 (**d**) Comparison of b-wave amplitude at 10 mcds/m^2^ (left panel) and latency (right panel). (**e**) Scotopic a-waves in response to single flash of 10 cds/m^2^ in *Ntn-4*^−/−^ (red) and WT (black) at P28 (**f**) Comparison of a-wave amplitude at 10 mcds/m^2^ (left panel) and latency (right panel). (**g**) Scotopic b-waves in response to single flash 10 mcds/m^2^ in *Ntn-4*^−/−^ (red) and WT (black) at P28 (**h**) Comparison of b-wave amplitude at 10 mcds/m^2^ (left panel) and latency (right panel). All recordings: N = 8 eyes of 4 mice; *p < 0.05; **p < 0.01.

Next to test if the faster revascular response after OIR in *Ntn-4*^−/−^ mice had a functional consequence, scotopic ERG was performed on wild-type and *Ntn4*^−/−^ mice (Fig. 3) using standard ISCEV conditions. ERGs were recorded at P17 and P28 (5 and 16 days after oxygen treatment, respectively). At P17, there were no differences in the a-wave parameters between the two mouse strains suggesting comparable photoreceptor function, whereas the b-wave latency increased in the *Ntn-4*^−/−^ mice (105.3 μV ± 7.6 μV, N = 8 versus 90.6 μV ± 6.4 μV, N = 8, p = 0.002) (Fig. 3a–d). At P28, the *Ntn-4*^−/−^ mice showed significantly increased a-wave (58.7 μV ± 44, N = 8, p = 0.012) and b-wave (458 μV ± 63 μV, N = 8, p = 0.006) amplitude, but still increased b-wave latency (88.5 ± 2.1, n = 8, p = 0.027) than the WT mouse (517 ms ± 55 ms, N = 8, 362 ms ± 40 ms, N = 8, and 83.3 ms ± 5.7 ms, N = 8, respectively) (Fig. 3e–h). Thus, the absence of netrin-4 accelerates at first the vasobliterative phase of OIR as well as the recovery from these effects. As shown in Suppl. Fig. 7 this functional difference is not due to a different baseline response in genetically different mice but due to a difference in OIR response.

### Differential regulation of netrin-4 mRNA expression at OIR compared to netrin-1 mRNA expression

**Figure 4:**
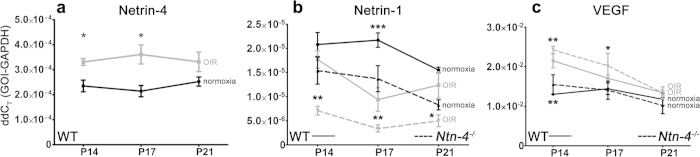
Differential regulation of netrin-4, netrin-1, and VEGF-A in OIR. (**a**) Comparison of netrin-4 mRNA expression in oxygen treated (grey) and untreated (black) WT mice. Netrin-4 expression is significantly higher at P14 and P17 after oxygen treatment, and decreases at P21 to normoxic level. (**b**) Comparison of netrin-1 mRNA expression in oxygen treated (grey) and untreated (black) WT (solid line) and *Ntn-4*^−/−^ mice (interrupted line). In contrast to netrin-4 and VEGF-A the mRNA expression of netrin-1 is lower after OIR and increases at P21 to normoxic level. (**c**) Comparison of VEGF-A mRNA expression in oxygen treated (grey) and untreated (black) WT (solid line) and *Ntn-4*^−/−^ mice (interrupted line). As expected, the maximal VEGF-A mRNA expression after OIR is at P14 and decreases at P21 to normoxic level. The expression pattern of VEGF-A is very similar in WT mice and *Ntn-4*^−/−^. All measurements N ≥ 3 to 5, *p < 0.05; **p < 0.01; ***p < 0.001.

We next analysed whether expression of netrin-4 is differentially regulated during pathologic angiogenesis. Expression of netrin-4 and netrin-1 were examined under normoxia and relative hypoxia at P14, P17, and P21 in WT mice using qPCR. Under normoxic conditions, netrin-4 mRNA expression was stable from P14 to P21 at a ΔΔC_T_ value of about 2.3 × 10^−04^, N = 3 (Fig. 4a, black). On the other hand, netrin-1 mRNA expression remained at a 10 times lower but also constant level of about 2.1 × 10^−05^ (P14: 2.08 × 10^−5^ ± 1.43 × 10^−6^; N = 3) between P14 and P17 in the WT, before declining further to 1.5 × 10^−05^ at P21 (Fig. 4b). In *Ntn-4*^−/−^ mice a similar expression pattern of netrin-1 mRNA was observed: Starting at P14 with a ΔΔC_T_-value of 1.54 × 10^−5^± 2.82 × 10^−6^ (N = 4) and decreasing to 8.3 × 10^−6^± 1.06 × 10^−6^; (N = 4) at P21. There is no compensating netrin-1 overexpression in *Ntn-4*^−/−^ mice due to the missing of netrin-4. OIR significantly increased the expression of netrin-4 mRNA at P14 (3.31 × 10^−4^ ± 1.34 × 10^−5^; N = 3 p = 0.024) and at P17 (3.61 × 10^−4^ ± 3.77 × 10^−5^; N = 4; p = 0.029). At P21, netrin-4 mRNA expression rates reached a similar level as in untreated retinas (3.32 × 10^−5^ ± 8.69 × 10^−6^; p = 0.191) (Fig. 4a, grey). There is an opposite reaction of the netrin-1 mRNA expression to OIR than of netrin-4 expression (Fig. 4b): In the WT retina (Fig. 4b, solid line), netrin-1 expression at P14 starts at the same level as at normoxia (1.77 × 10^−5^ ± 1.776×10^−6^; N = 3), but then decreases at P17 (0.94 × 10^−5^ ± 2.42 × 10^−6^; N = 4) and increases again to the normoxic level at P21 (1.24 × 10^−5^ ± 2.7 × 10^−6^, N = 5). In *Ntn-4*^−/−^,(Fig. 4b, interrupted line) netrin-1 mRNA expression decreases even more after OIR and is significant lower than at normoxia (P14: 7.1 × 10^−6^ ± 9,07 × 10^−7^; N = 3; p = 0.0049; P17: 3.4 × 10^−6^ ± 7,38 × 10^−7^; N = 3; p = 0.0015; P21: 5.0 × 10^−6^ ± 1.22 × 10^−6^; N = 3; p = 0.0122). Also after OIR there is no compensating netrin-1 mRNA overexpression in *Ntn-4*^−/−^.

### Differential VEGF-A mRNA expression after OIR in *Ntn4* ^−/−^ mice

It is well established that VEGF is a fundamental regulator of normal and abnormal angiogenesis^25^. Therefore, mRNA levels of VEGF-A expression were measured in oxygen treated and untreated WT and netrin-4 knock-out mice (Fig. 4c). Under normoxic conditions, VEGF-A mRNA expression in WT (solid line) was constant from P14 to P21: 1.3 × 10^−2^ ± 4.84 × 10^−4^ at P14, 1.44 × 10^−2^ ± 2.54 × 10^−3^ at P17, and 1.18 × 10^−2^ ± 2.69 × 10^−3^ at P21 (N = 3 for each time point). A similar and constant VEGF-A mRNA expression was also noted in *Ntn-4*^−/−^ (interrupted line) in normoxia: P14: 1.55 × 10^−2^ ± 2.47 × 10^−3^, P17: 1.41 × 10^−2^ ± 2.29 × 10^−3^, P21: 1.02 × 10^−2^ ± 2.07 × 10^−3^ (N = 4 for all time points). After oxygen treatment, VEGF-A mRNA expression in both genotypes (WT: solid line, *Ntn-4*^−/−^: interrupted line) increased significantly as expected and had a maximum at P14 (WT: 2.15 × 10^−2^ ± 3.04 × 10^−3^; N = 3 and *Ntn-4*^−/−^: 2.42 × 10^−2^ ± 0.91 × 10^−3^, N = 3). Also in both genotypes VEGF-A expression decreased at P17 (WT: 1.71 × 10^−2^ ± 1.71 × 10^−3^; N = 4 and *Ntn-4*^−/−^: 2.02 × 10^−2^ ± 3.17 × 10^−3^; N = 3) thereafter reaching the normoxic level at P21 (WT: 1.35 × 10^−2^ ± 1.52 × 10^−3^; N = 5 and *Ntn-4*^−/−^: 1.34 × 10^−2^ ± 1.53 × 10^−3^; N = 3)

Thus, irrespective of the absence of netrin-4, VEGF-A mRNA expression follows its normal course in the OIR model.

### Activation of netrin receptor expressing glia cells after OIR

**Figure 5:**
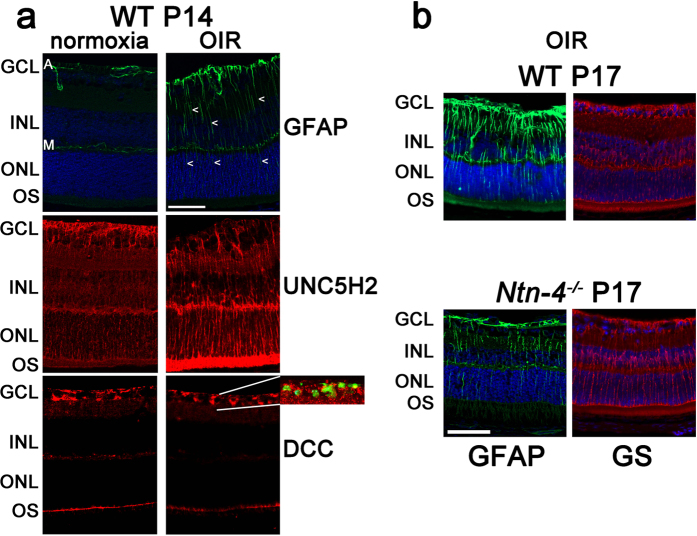
Activation of GFAP and netrin receptor expressing glia cells after OIR. (**a**) Immunodetection in sections of GFAP (green) and the netrin receptors UNC5H2 or DCC (red) from WT retinas at P14 at normoxia (left panel) and after OIR (right panel). The patterns of staining are consistent with expression of these two receptors by both Müller cells and astrocytes. Astrocyte extensions in the GCL are marked “A”. Müller cell extensions in the outer plexiform layer are marked “M”. Cell extensions in the inner and outer nuclear layer are marked by arrowheads.The uniform localisation of DCC in astrocytes at normoxic conditions is irregular and spotty. Insert shows a detail of the co-staining. The positive staining in the outer segments (OS) of the photoreceptors is due to unspecific binding of the secondary antibody. Scale bar indicates 50 μm. (**b**) GFAP staining of WT and *Ntn-4*^−/−^ retinal sections after OIR. GFAP stains astrocytes and Müller cells. Activation of astrocytes and Müller cells looks similar in both *Ntn-4*^−/−^ and WT. Nuclei were stained with DAPI. Bar scale indicates 50 μm.

As demonstrated above, differential regulation of netrin-4 has a role in the stability of mature blood vessels and is the prerequisite for pathologic blood vessel formation in OIR. We now aimed to identify which retinal cell type is able to react to changes of netrin-4 in the basement membrane. Although netrin-4 receptors are not well established, the expression of cognate receptors of other netrin family members was investigated. By RT-PCR and Western Blot analysis, Liu and colleagues have recently shown that UNC5B (=Unc5H2), UNC5C, UNC5D, DCC, neogenin, and A2b are all expressed in the retinas of mice^26^. But only Unc5H2 was upregulated after OIR. We therefore concentrated on Unc5H2 and only on one of the non-reacting receptors (DCC) to identify their cellular location. In normoxia at P14, glial fibrillary acidic protein (GFAP) is staining prominently astrocytes. On the other hand, after OIR at P14 and P17, astrocytes and Müller cell hyperplasia can be detected by GFAP and glutamine synthetase (GS)^27^, indicating gliosis that involves both astrocytes and Müller cells (Fig. 5). Immunostaining for both Unc5H2 and DCC showed widespread staining throughout the retina. The patterns of staining are consistent with expression of these two receptors by both Müller cells and astrocytes (Fig. 5). After OIR, Unc5H2 is localized in radial fibres running throughout the ONL, a pattern which is more distinctly attributable to up-regulation by Müller cells (Fig. 5a, right panel) under these conditions compared to normoxia (Fig. 5a, left panel). In normoxic retina, DCC immunoreactivity in the GCL appears to co-localize with GFAP suggesting expression in astrocytes (Fig. 5a, left panel), and these pattern does not change in relative hypoxia (Fig. 5a, right panel). Thus it is likely that Müller cells or astrocytes in the inner retina can respond to changes in netrin-4 expression in the basal membrane.

GFAP and GS staining of retinal sections after OIR at P14 and P17 reveals GFAP localisation in astrocytes and Müller cells (Fig. 5a,b). Astrocyte and Müller cell hyperplasia is similar in both *Ntn-4*^−/−^ and WT mice (Fig. 5b).

### Reduced leakage area but similar scar area in the choroid of *Ntn-4* ^−/−^ mice after laser photocoagulation

Given that netrin-4 is differentially regulated under oxygen influence and *Ntn-4*^−/−^ mice exhibited an altered blood vessel growth in the ROP model, it is likely that netrin-4 plays a general role in blood vessel homeostasis or pathological angiogenesis. To further explore a more general regulatory role of netrin-4 in neo-angiogenesis, we investigated laser-induced CNV in both WT and *Ntn-4*^−/−^ mice.

**Figure 6:**
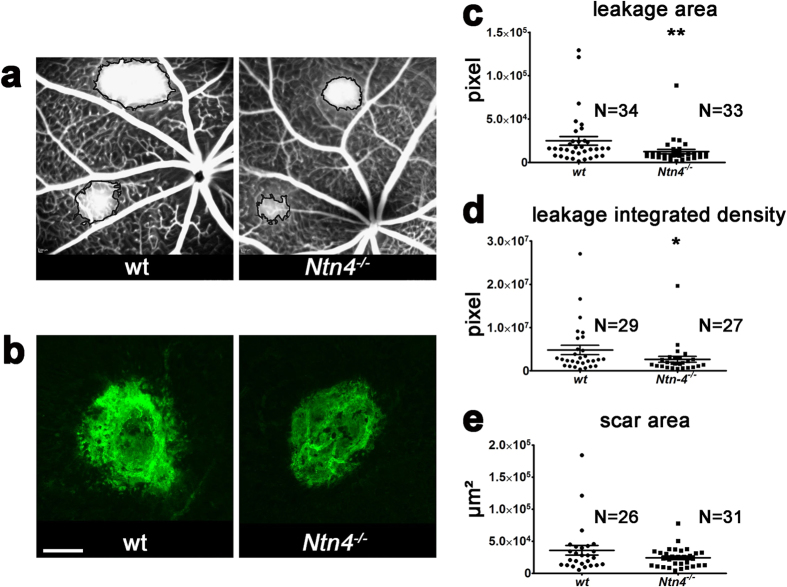
Lower leakage in *Ntn-4*^−/−^ mice two weeks after laser photocoagulation but no different scar area. (**a**) SLO angiography of vessel leakage area two weeks after laser photocoagulation in WT and *Ntn4*^−/−^. There is a smaller leakage area (**c**) and integrated density (**d**) in *Ntn4*^−/−^ mice. (**b**) Isolectin IB4 stained retinal flat mounts of laser scar area two weeks after laser photocoagulation in WT and *Ntn4*^−/−^ mice. There is no difference in the laser scar area (**e**) between WT and *Ntn4*^−/−^ mice. N is the number of leakage area, leakage integrated density, or scar area and came from three mice, each. Scale bar indicates 100 μm. *p < 0.05, **p < 0.01.

One and two weeks following laser photocoagulation, fluorescein leakage from laser scars was quantified as a measure of new blood vessel formation. Leakage area as well as leakage integrated density measured in *Ntn-4*^−/−^ mice and WT mice were significantly different two weeks after photocoagulation (*Ntn-4*^−/−^, 2.641×10^6^ pixel ± 701031, n = 27 vs WT, 4.805×10^6^ pixel ± 106300; n = 29, p = 0.0358) (Fig. 6). In contrast, no significant differences were observed one week (*Ntn-4*^−/−^, 3.985×10^6^  ± 696513 pixel; n = 27 vs WT, 4.296×10^6^  ± 873013 pixel; n = 19, p = 0.6717) after laser treatment. In contrast to the effect on vascular re-growth, there was no difference in the area of scar formation. Isolectin-IB_4_ staining in sclera flat-mounts revealed no significant difference (p = 0.2795) in the size of the laser scar area between the groups (24210 μm ± 2730; n = 31, *Ntn-4*^−/−^ vs 35824 μm ± 7536; n = 26, WT) (Fig. 6) two weeks after laser photocoagulation. These data indicate that netrin-4 has a minor impact in laser-induced choroidal neovascularization, a model in which neovascularization is strongly driven by inflammation and not by hypoxia.

## Discussion

Using *Ntn-4*^−/−^ mice, we demonstrate that differential expression of netrin-4 plays a role in pathophysiology of neovascularization and influences ischemia related but not inflammatory neovascularization. Analysis of the temporal and spatial distribution of netrin-4 expression demonstrated that netrin-4 is anti-angiogenic and enriched in adult blood vessel basal lamina to maintain blood vessel stability.

During postnatal development of the WT retina, netrin-4 and netrin receptors are differentially expressed depending on the developmental status of the vasculature. Close to the time-point of blood vessel maturation, netrin-4 staining was found throughout the retinal vasculature up to the capillary level, but was more prominent in superficial layers of the arterial side in the vascular tree, albeit not in the deeper layers. These findings on a protein level are in line with the progression of netrin-4 mRNA-expression during vessel development. A constant netrin-4 mRNA levels are found at the late phase of retinal vessel formation (P14–P21). In accordance, analysis of retinal vascularization in netrin-4 deficient mice revealed that vessels and capillaries developed normally. Thus, netrin-4 expression is not associated with the early stages of postnatal blood vessel development, but instead with the termination of their development and is likely to be involved in stabilizing mature blood vessels. This implies, however, that there is no active role for netrin-4 in normal vascular development but is involved with neovascular events as predicted by Li *et al.*^12^.

Analysis of potential netrin-4 receptors also revealed a possible functional correlation with a role in stabilisation. Although it is not fully established that netrin-4 binds to the known receptors of the netrin family (e.g. Unc5H2 and DCC), data obtained here suggest that retinal glial cells, Müller cells and astrocytes, express these proteins indicating that netrin-4 plays a role in mediating interactions between the vascular and the neuronal system, as is assumed for the other members of the netrin protein family. This result is a good starting point for further exploring neurovascular coupling in *Ntn-4*^−/−^ retina undergoing vascular pathogenesis scenarios.

In contrast, to the minor role during retinal development, netrin-4 expression may, however, have a major impact on pathological blood vessel formation. The role of netrin-4 in hypoxia-driven pathologic neovascularization in the OIR model was investigated in *Ntn-4*^−/−^ and WT mice. After OIR the avascular zone was more rapidly closed in *Ntn-4*^−/−^ mice as compared to WT. Moreover, in this sequel, the *Ntn-4*^−/−^ retina showed faster functional recovery after OIR. At P17 rod function was similar in *Ntn-4*^−/−^ and WT. On the other hand, *Ntn-4*^−/−^ mice displayed reduced functionality of bipolar and Müller cells at this time point. In contrast, at P28 *Ntn-4*^−/−^ mice displayed higher rod activity and comparable bipolar and Müller function to WT. However, the faster functional recovery in knock-out mice may not be solely due to vascular normalization. Directly improving neuronal cells functions cannot be ruled out, although it is difficult to separate them due to the intensive interaction between the nerve system and the vascular system.

Thus, absence of netrin-4 accelerates the hypoxia-driven blood vessel regression during the time of oxygen exposure but also the recovery from OIR, indicating netrin-4 is an anti-angiogenic factor. The latter conclusion was supported by analysing of time-dependent netrin-4 expression in OIR. After oxygen exposure, an increase in netrin-4 expression was observed at the mRNA at P17, as compared to controls. Since P17 in OIR is the period of highest pathological angiogenic activity, up-regulation of netrin-4 is likely one of the prerequisites for maturing vessels and thus normalisation of angiogenesis. Therefore, in the *Ntn-4*^−/−^ mice the complete absence of netrin-4 permits the faster recovery of the avascular area after OIR by allowing the faster sprouting of new and outgrowth of immature vessels, because maturation is delayed in this genotype.

The increased vessel loss in *Ntn-4*^−/−^ mice at P10 may suggest that netrin-4 has an anti-apoptotic function. To date, only for netrin-1 in combination with its receptor UNC5B there is an anti-apoptotic function described^28^. In a tumor model, netrin-4 overexpression was shown to inhibit tumor growth. But, although this is associated with decreased tumor cell proliferation and enhanced tumor cell apoptosis *in vivo*, the authors could not show a direct effect on tumor proliferation and tumor apoptosis *in vivo* and *in vitro*^29^.

Bucher *et al.* 2013 found apoptosis in astrocytes during the hyperoxic phase between P8 and P10 in mice expressing H2B-GFP (green fluorescent protein fused to histone 2B) from the endogenous *Pdgfra* promoter^30^. Also retinal thinning in hypovascular and avascular regions were found in mice after OIR^31^, but it was not clear whether this occurred due to arrested retinal development and/or ischemia induced apoptosis. The possible involvement of netrin-4 with apoptosis would be an interesting topic for a further analysis of its function in the eye.

Analysis of time-dependent VEGF-A expression in *Ntn-4*^−/−^ further supports the idea that netrin-4 expression plays a role in establishing an anti-angiogenic environment. During physiological postnatal development, expression of both netrin-4 and VEGF-A is constant from P14 to P21. As postulated earlier, given that netrin-4 is potentially anti-angiogenic, its high level of expression may counterbalance a high VEGF-A expression levels and stabilise the mature vessels.

After oxygen challenge, the expression dynamics of netrin-4 and VEGF-A are quite similar. VEGF-A expression peaks at a time point (P14) when netrin-4 expression is also high. Thus, in OIR both VEGF-A and netrin-4 show a high and balanced expression to help normalizing angiogenesis. Netrin-4 may have a specific anti-angiogenic function as Netrin-1 does not show any compensatory regulation in *Ntn4*^−/−^ mice.

Netrin-4 is required for maintenance of mature blood vessels and its expression needs to be balanced with the expression of VEGF in physiological as well as pathological angiogenesis.

Actually, in *Ntn-4*^−/−^ retinas, the pathological vessels at P17 showed partly a different morphology than the WT. In *Ntn-4*^−/−^, some neovascular vessels appear as large branched pathological vessels compared to the more circumscribed neovascular tufts in WT, with decreased cell numbers but with similar tuft area; highlighting a possible altered proliferation activity of endothelial cells during angiogenesis when netrin-4 is absent but VEGF-A level is still high. The similar pericyte distribution in neovascular tufts of WT and *Ntn-4*^−/−^ indicates no direct influence of Netrin-4 on the interaction of pericyte and endothelial cells.

As discussed above, netrin-4 plays a role in physiological vascular stability and in the recovery of pathologic neovascularization after hyperoxic challenge. In order to analyse whether netrin-4 has a more general role in the regulation of neo-angiogenesis, we employed another model for neovascularization, the laser-induced CNV in both WT and *Ntn-4*^−/−^ mice. The finding that the leakage area and the integrated leakage density of *Ntn-4*^−/−^ mice was significantly increased (as compared to WT mice) only two weeks after photocoagulation but with no associated differences in the laser area, indicates a minor role for netrin-4 in laser-induced choroidal neovascularization^14^ , a model in which neovascularization is strongly driven by inflammation rather than hypoxia.

The temporal and spatial expression pattern of netrin-4 and its receptors in relation to VEGF-A expression together with the faster recovery after oxygen challenge in *Ntn-4*^−/−^ mice allow us to postulate a model of netrin-4 action in the retina. Results from the OIR model, in accordance with previous work *in vitro*^19^, suggest expression of netrin-4 is regulated by changes in oxygen tension. Furthermore, we suggested both Müller cells and astrocytes as sites of DCC and Unc5H2 expression, which would enable interaction with netrin-4 positive basement membranes. Again, although the cognate receptors for netrin-1 are not yet established as receptors for netrin-4, their expression in Müller cells and astrocytes and perhaps retinal neurons, propose they have an interacting role in retinal angiogenesis. We postulate that smooth muscle and endothelial cells are the major source for netrin-4, as reported previously^6^. Taken these key observations together, we think that these cells enrich the basal membranes with netrin-4 to stabilise the mature vessels at the end of their development.

The hypothesised role of netrin-4 role in vessel stabilization and oxygen-tension dependent vascular homeostasis aligns well with observations regarding a broader role of netrin-4 in disease, especially cancer^32^,^29^. Netrin-4 has been identified as a prognostic marker for tumours^33^ and exhibits anti-tumorigenic potential, albeit through unknown mechanisms^13^,^34^. Netrin-4 application was shown to decrease intracellular signalling related controlling proliferation, but it could neither be linked to a specified cell type nor to a direct pathway^13^,^34^. Since tumour growth, in particular, depends on oxygen-driven blood vessel growth, we suggest that the netrin-4-dependent regulation of blood vessel stability explains these observations.

## Material and Methods

### Animals

All animal experiments were adhered to the ARVO Statement for the Use of Animals in Ophthalmic and Vision Research and were approved by governmental Animal Care and Use Committees (LaGeSo, Berlin).

Mice were not littermates. C57/Bl6J mice were purchased from Charles River (Wilmington, MA, USA) or Janvier (Cedex, France). *Ntn-4*^−/−^ mice were generated as described^12^ and are on a C57BL/6J-background. The genotype was determined by PCR analysis of genomic DNA prepared from tail or ear samples. Primers used: wild type (WT) and null allele forward AGCAGCCTTTAAACATCCTGAG, WT allele reverse GAAAGCTCCGGGCAGACACTATGTG, and null allele reverse CAAATGTGTCAGTTTCATAGCC.

Animals were fed regular laboratory chow and water ad libitum. A 12-hour day–night cycle was maintained.

### Mouse Model of Oxygen-Induced Retinopathy

To induce reproducible proliferative retinal neovascularization in retinopathy of prematurity, the protocol by Smith *et al.*^35^) was performed under modified conditions^36^. Mice - the nursing dam and her new-born pups were housed in room air conditions from postnatal day P0 to P7. On P7, the animals were exposed to 75% oxygen for 5 days and then, transferred back to room air. A special oxygen chamber (Model THF3384) was used (EHRET Labor- u. Pharmatechnik GmbH, Emmendingen, Germany). Surrogate nursing dams were provided if mother died - a rare incident. Control mice were maintained in room air. Pups were removed from their cages for retinal analysis on days P10, P12, P14, P17, and P21. All experiments were performed at least in triplicate for each time point. Only animals with a weight above 5 g at P14 were included in the study^37^.

### Laser-induced CNV Model and fluorescence angiography

To investigate the role of netrin-4 in choroidal neovascularization (CNV), adult WT and *Ntn-4*^−/−^ mice underwent laser exposure^38^.

An argon laser (Visulas 532 s, Carl Zeiss Meditec, Oberkochen, Germany) was used to set four to five distinct 50 μm wide spots (120 mW intensity, 100 ms duration with a wavelength of 532 nm) around the optic disc. Rupture of Bruch’s membrane was indicated by appearance of a bubble at the site of photocoagulation^39^. After two weeks, the treated eyes were investigated by means of a fluorescence angiography in order to evaluate leakage from the nascent pathological choroidal blood vessels. We followed the method developed by Ryan^40^, adapted for mice^41^. The animals were intraperitoneally injected with 200 μl 5% fluorescein sodium (Alcon Pharma GmbH, Freiburg, Germany) and placed in front of the ophthalmoscope (Spectralis OCT, Heidelberg Engineering, Heidelberg, Germany). Images were analysed using ImageJ (NIH, USA) having into account leakage area and integrated density.

### Visualization and Quantification of Retinal Vascularization in Whole-Mounts

Eyes were enucleated and fixed in 4% PFA solution for 15 minutes. The cornea was dissected with a circumferential limbal incision, followed by removal of the lens and vitreous. Four radial cuts were made to allow flattening and subsequently, retina was isolated by sectioning the optic nerve. Retinas were repeatedly washed and incubated in a blocking solution containing normal goat serum. Vasculature was detected and analysed using Alexa 488-conjugated *GS* Isolectin-IB_4_ (1:200; Thermo Fisher Scientific (Invitrogen), Waltham MA) as described^38^ (Table 1). Pericytes were detected using an antibody against NG2 (Table 1), a commonly extended marker^42^. Netrin-4 (Table 1) was detected according to Cellerino *et al.*^43^.

All retinas were mounted onto glass-slides and subjected to microscopy imaging with a standardised technique to compare vascular density and to analyse areas of neovascularization. Microscopes employed were MZ FLIII (Leica Microsystems, Bensheim, Germany) with a charge-coupled device (CCD) camera (C4742-95-12ER; Hamamatsu, Hamamatsu City, Japan) and Axio Imager M2 fluorescence microscope (Zeiss, Göttingen, Germany).

Analysis was run using the soft wares OpenLab (Improvision Inc., Lexington, MA, USA) and ZEN 2012 (Zeiss, Göttingen, Germany). The total avascular area (in pixels) was then determined and normalised to the total retina area and the percentage of the non-vascularized area was calculated using ImageJ (NIH, USA). All retinas were analysed in a masked fashion to minimise sampling bias.

### Histology and immunohistochemistry on paraffin sections

Animals were sacrificed at settled time points, and eyes fixed in Methacarn (60% methanol, 30% 1,1,1 -trichlorethane, 10% acetic acid) overnight at room temperature (RT) or 4% PFA overnight at 4 °C and routinely processed for paraffin embedding. Sections (5 μm) were used for immunohistological analysis. Some paraffin sections were subjected to standard hematoxylin-eosin (HE) staining for retinal structural analysis.

Immunodetection was performed on deparaffinised sections. Different antigen retrieval processes were applied before incubation with a blocking solution containing 5% BSA. Blood vessels were stained with *GS* Isolectin-IB_4_. Müller cell gliosis was detected via Glial Fibrillary Acidic Protein (GFAP) and via Glutamine synthetase (GS). Netrin-4 and its receptors Deleted in Colorectal Carcinoma (DCC) and Unc-5 Homolog 2 (Unc5H2) specifications are in Table 1. All primary antibody incubations were performed overnight at 4 °C, but for anti-DCC and anti-Unc5H2, which incubation was extended up to 4 nights. After three washing steps with TBS, species-appropriate fluorescence-labelled secondary antibodies were applied for 1 h at RT (Table 2). Sections were mounted in Fluorescence Mounting Medium (DAKO, Hamburg, Germany) and examined using the Axio Imager M2 fluorescence microscope (Zeiss, Göttingen, Germany).

### RNA Isolation and RT-PCR

Freshly isolated retinas were stored in RNAlater solution at −20 °C until RNA isolation and then homogenized in lysis buffer (RNeasy Kit, Qiagen, Hilden Germany). RNA isolation and cDNA synthesis was performed according to the manufacturer’s instructions (Qiagen, Hilden, Germany). Expression levels of 6 calibration genes were measured in WT mice retinas, using the geNorm calculation tool V3.3, based on the recommendations of Vandesompele *et al.*^44^. Because GAPDH was found to be one of the most stable genes in our experiments, it was used to normalise gene expression levels.

The mRNA expression levels of VEGF-A, netrin-1, netrin-4, and GAPDH in the mouse retinas from different strains and experimental conditions were measured in triplicate by Real Time RT-PCR using the Rotor-Gene SYBR

Green PCR Kit (Qiagen, Hilden, Germany) on a Rotor-Gene Q (Qiagen, Hilden, Germany). Primer sequences are collected in Table 3. The analysis was repeated three times. To verify specific amplification, PCR products were subjected to melting curve analysis. Genomic DNA contamination was excluded by choosing primers hybridizing to different exons or spanning exon borders. Moreover, control amplification reactions that were performed with non-transcribed RNA as templates gave only background fluorescence. Quantification of the calibrated target genes was carried out using the comparative CT (threshold cycle, ΔΔC_T_) method using Rotor-Gene Q software 2.2.3 (Qiagen, Hilden, Germany) as described^45^. Only animals with comparable mean body weight per group at the different time points were analysed (Table 4). There is no significant body weight difference between WT mice and *Ntn-4*^−/−^ mice when comparing mice held at normoxia or treated with oxygen with the exception of P21 mice after OIR.

### Electroretinography (ERG)

Mice were dark-adapted overnight, pupils were dilated by tropicamide 0.5% and atropine 1%, and mice were anesthetised by injection of xylazine (15 mg/kg body weight) and ketamine (100 mg/kg). The animals were positioned on a warming table to maintain body temperature. A monopolar contact lens electrode was used as recording electrode (for both eyes) and a subcutaneous fixed platin needle was the reference and ground electrode. For recording, the mouse was placed into a commercially available Ganzfeld bowl (Roland Consult, Brandenburg, Germany).

In the dark-adapted state, a flash series consisting of 10 steps started at –4.0 log cd • s/m−2 and reached 0.48 log cd • s/m−2. The signal was amplified by 10.000 with a band pass filter from 1 to 300 Hz. Oscillatory potentials were obtained with flash Intensity 0.48 log cd s/m−2 by band pass filtering from 100 to 500 Hz.

C-wave recordings were taken in the dark-adapted state, immediately after the scotopic ERG. Stimulus duration was 250 ms. Stimulus (green light) energy levels used were 0.48, 0.95 and 1.25 log cd • s/m−2 with band pass filtering from 0.1 to 30 Hz.

For a-wave recording, 3 additional flash energies were applied: 0.97, 1.48 and 1.97 log cd s/m−2.

After recording the a-wave, the background light (1.8 log cd m−2) was turned on, the animals were further light-adapted for 10 minutes, and the photopic ERG was recorded using a series of flash energies (0.7 and 1.3 log cd • s/m−2; band pass filtering 1-300 Hz; average of 10 recordings at 1.5 Hz).

## Statistical Analysis

All results are expressed as the mean ± SEM. After testing for normal distribution (Shapiro–Wilks), the data were compared by unpaired T-test if the Levenne-test showed equal variance. Otherwise a non-parametric test (Whitney-Mann-U) was used. For ERG a Kruskal–Wallis test for independent samples was used. For qPCR-analysis a power analysis (GPower 3.1.7, Franz Faul, University of Kiel) confirmed that with the used data and SEM a sample size of 3 is enough for a power >0.95. Differences were considered to be statistically significant when P values were less than 0.05.

## Additional Information

**How to cite this article**: Kociok, N. *et al.* Lack of netrin-4 modulates pathologic neovascularization in the eye. *Sci. Rep.* **6**, 18828; doi: 10.1038/srep18828 (2016).

## Supplementary Material

Supplementary Information

## Figures and Tables

**Table 1:** Primary Antibodies for Immunodetection.

Antibody	Retrieval treatment	Concentration	Source
Rabbit anti-DCC	Citrate Buffer	1:250	M. Koch
Rabbit anti-GFAP	Proteinase K	1:250	Dako
Rabbit anti-GS	Proteinase K	1:500	abcam
*GS* Isolectin-IB_4_ (AF488-conjugated)	None	1:200	Invitrogen
Rabbit anti-NG2 (Cy3-conjugated)	None	1:200	Millipore
Rabbit anti-Netrin-4	None	1:200-1000	M. Koch
Rabbit anti-Unc5H2	Citrate Buffer	1:500	M.Koch

**Table 2:** Secondary Antibodies for Immunodetection.

Antibody	Concentration	Source
Donkey anti-Rabbit (AF488-conjugated)	1:10000	Invitrogen
Goat anti-Rabbit (Cy3-conjugated)	1:5000	Dianova

**Table 3:** Primer sequences.

Gene	Forward Sequence (5′ → 3′)	Reverse Sequence (5′ → 3′)
Netrin-1	CCCTTGCATCAAGATTCCTGT	GAGTCACAGTCTTCCGGTTCC
Netrin-4	CCGCAGGCTTGAATGGAGTA	TATCACACTTGGGCTGCCG
VEGF-A^164,120,188^	CAGCTATTGCCGTCCGATTGAGA	TGCTGGCTTTGGTGAGGTTTGAT

**Table 4:** Body weight (g) of mice used for qPCR.

	C57BL6/J		*Ntn-4*^−/−^	
	Normoxia	OIR	Normoxia	OIR
P14	6.8 ± 0.5; N = 3	5.9 ± 0.3; N = 3	7.7 ± 1.2; N = 4	6.2 ± 0.3; N = 3
P17	7.8 ± 0.2; N = 3	7.5 ± 1.0; N = 4	8.5 ± 0.6; N = 4	7.0 ± 0.2; N = 3
P21	9.1 ± 0.6; N = 3	9.8 ± 0.6; N = 5; p = 0.009	10.2 ± 0.7; N = 4	8.1 ± 0.5; N = 3; p = 0.009
