# Supplementary material for: Lack of netrin-4 modulates pathologic neovascularization in the eye
Source: Sci Rep. 2016 Jan 6;6:18828. doi: 10.1038/srep18828 (PMC4702134; doi:10.1038/srep18828)
Supplement: Supplementary Information [file srep18828-s1.doc]

Supplementary information to manuscript (**SREP-15-23814A)**:

**Lack of netrin-4 modulates pathologic neovascularization in the eye**

Authors:

Norbert Kociok^1a^, Sergio Crespo-Garcia^1a^, Yong Liang^1,2^, Sabrina V. Klein^1^, Christina Nürnberg^1^, Nadine Reichhart^1^, Sergej Skosyrski^1^, Eva Moritz^3^, Anna-Karina Maier^1^, William J. Brunken^4^, Olaf Strauß^1^, Manuel Koch^3^, and Antonia M. Joussen^1*^


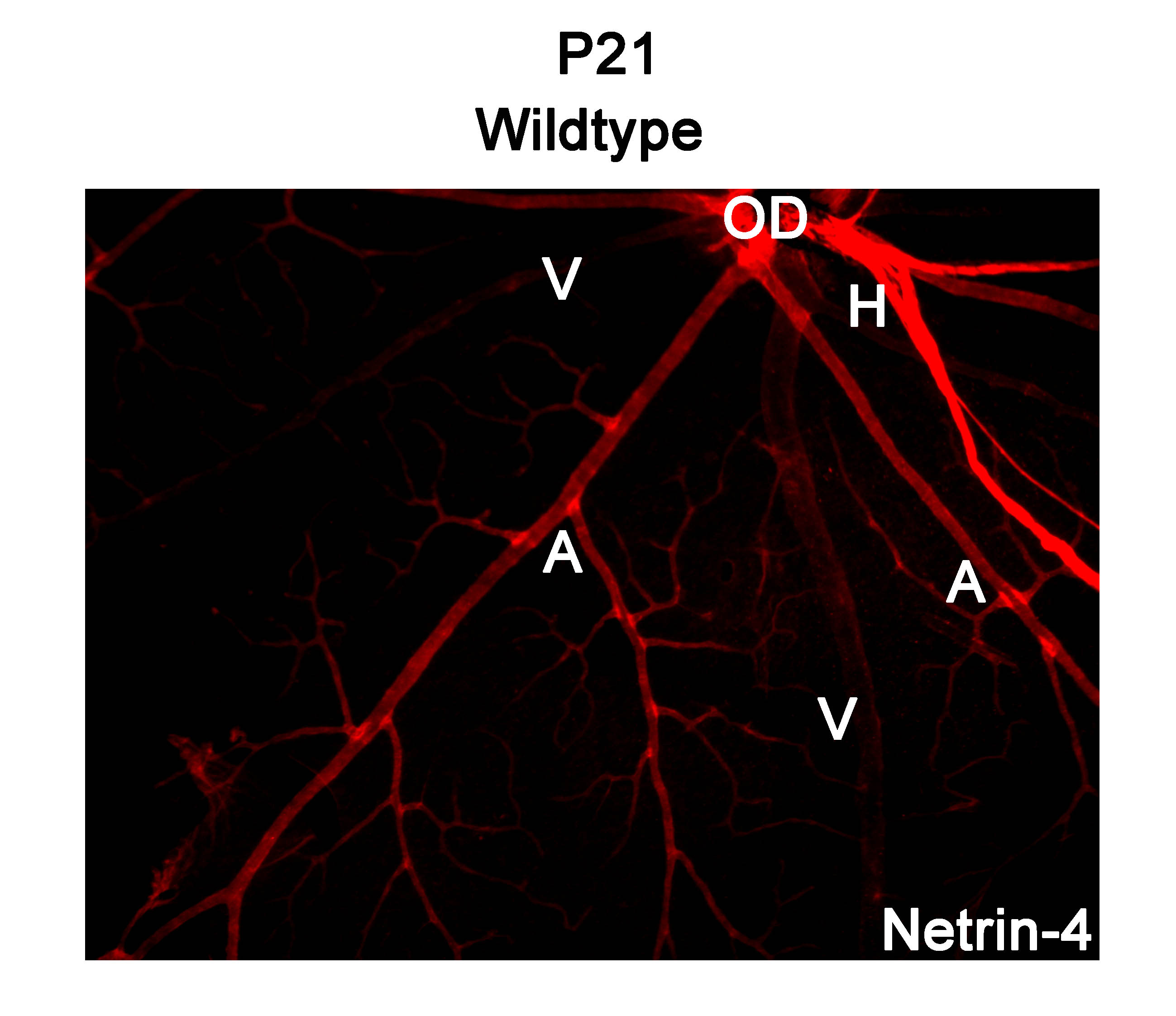


**Suppl. Fig. 1: Netrin-4 staining of a retinal flat mount at P21 of a WT mice.**

Netrin-4 staining is more intensive in arteries (A) than in veins (V).
H = hyaloid artery, OD = optic disc.


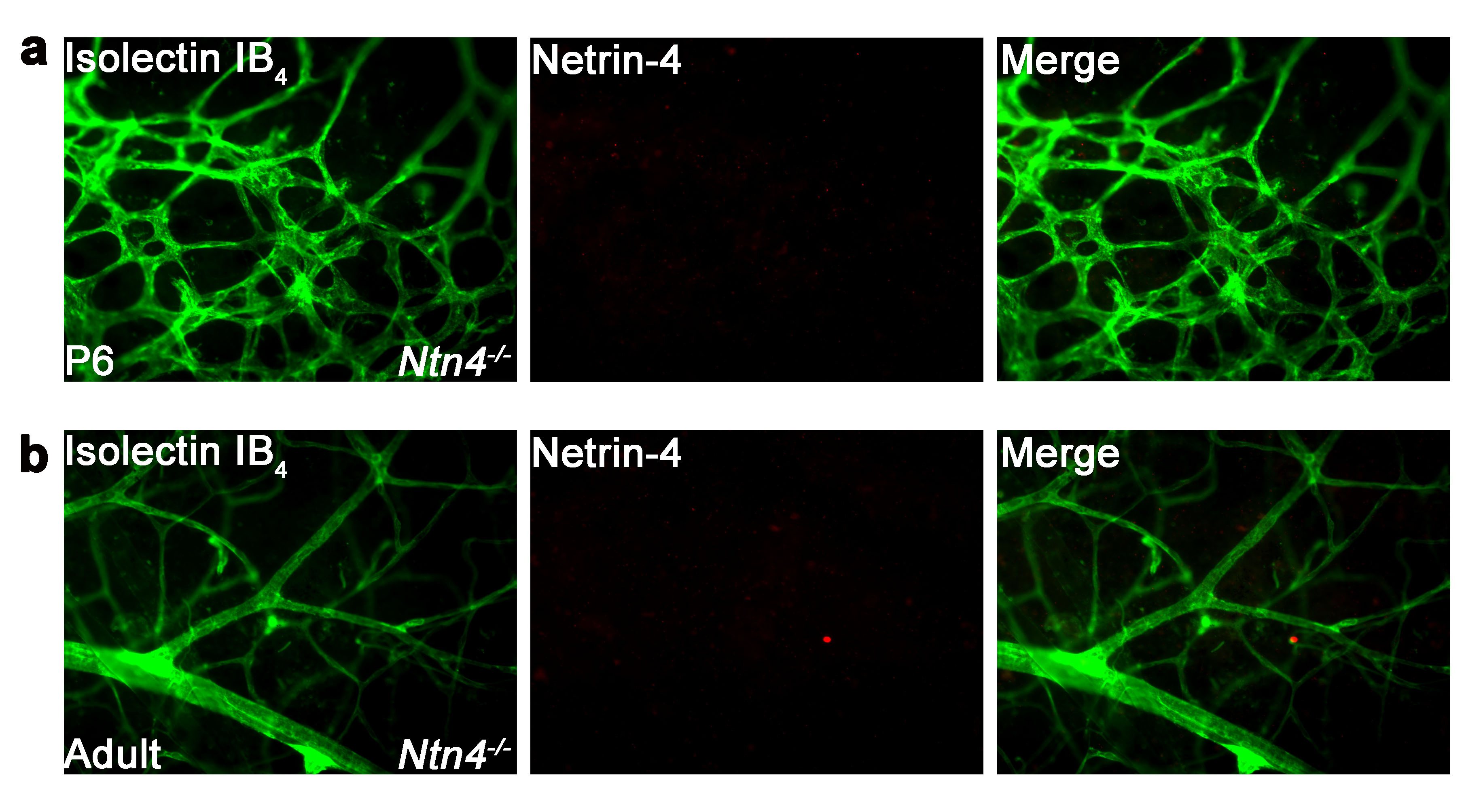


**Suppl. Fig. 2: Isolectin IB_4_ and Netrin-4 staining of retinal flat mounts on Ntn-4^-/-^ mice at normoxia**

Negative staining of Netrin-4 (red) demonstrates the absence of Netrin-4 in Ntn-4^-/-^ mice at P6 (a) and in adults (b).


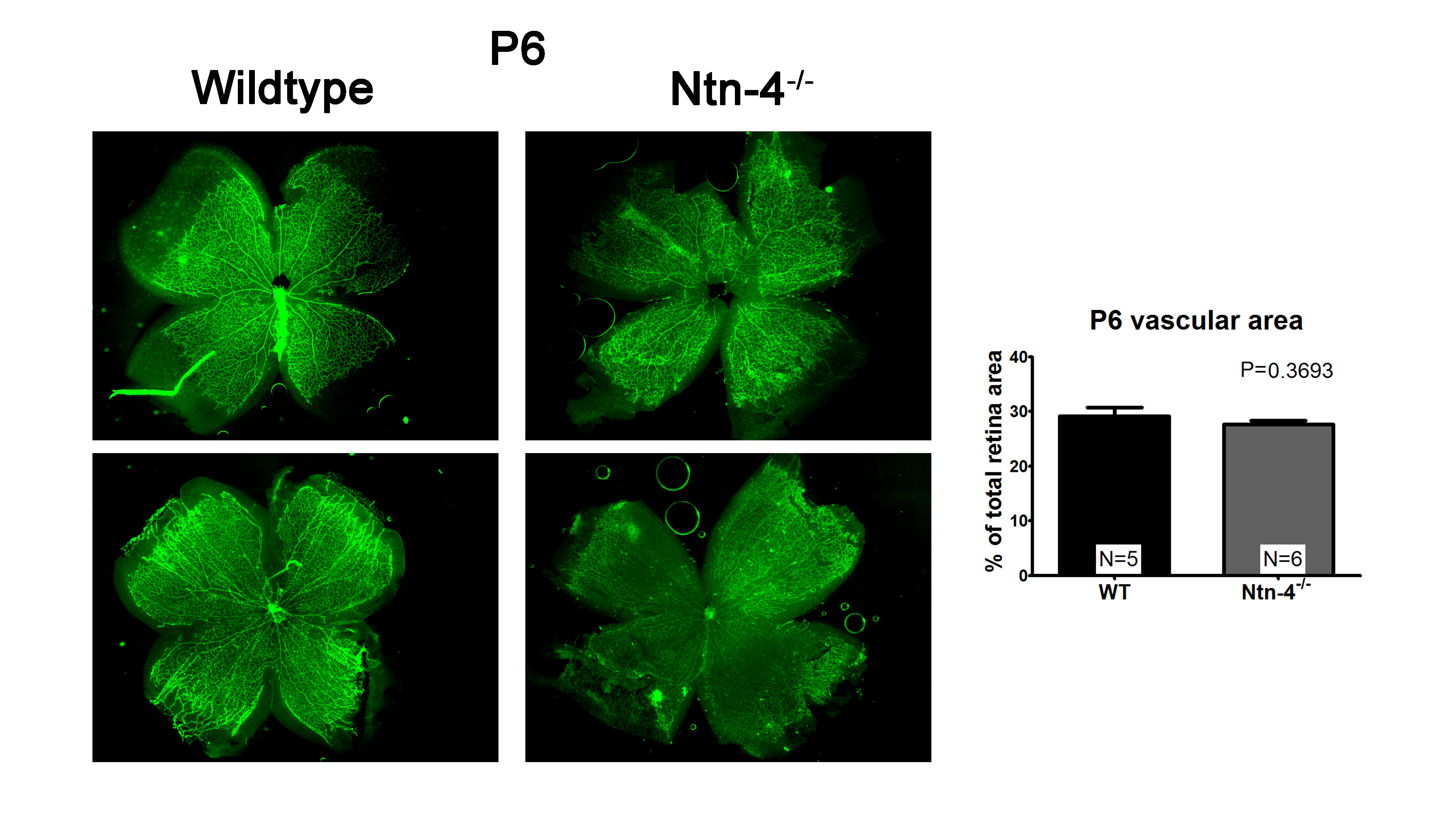


**Suppl. Fig. 3: Vascular area at P6 in WT and Ntn-4^-/-^ mice**

There is no difference in the vascular area at P6 between WT and Ntn-4^-/-^ mice.


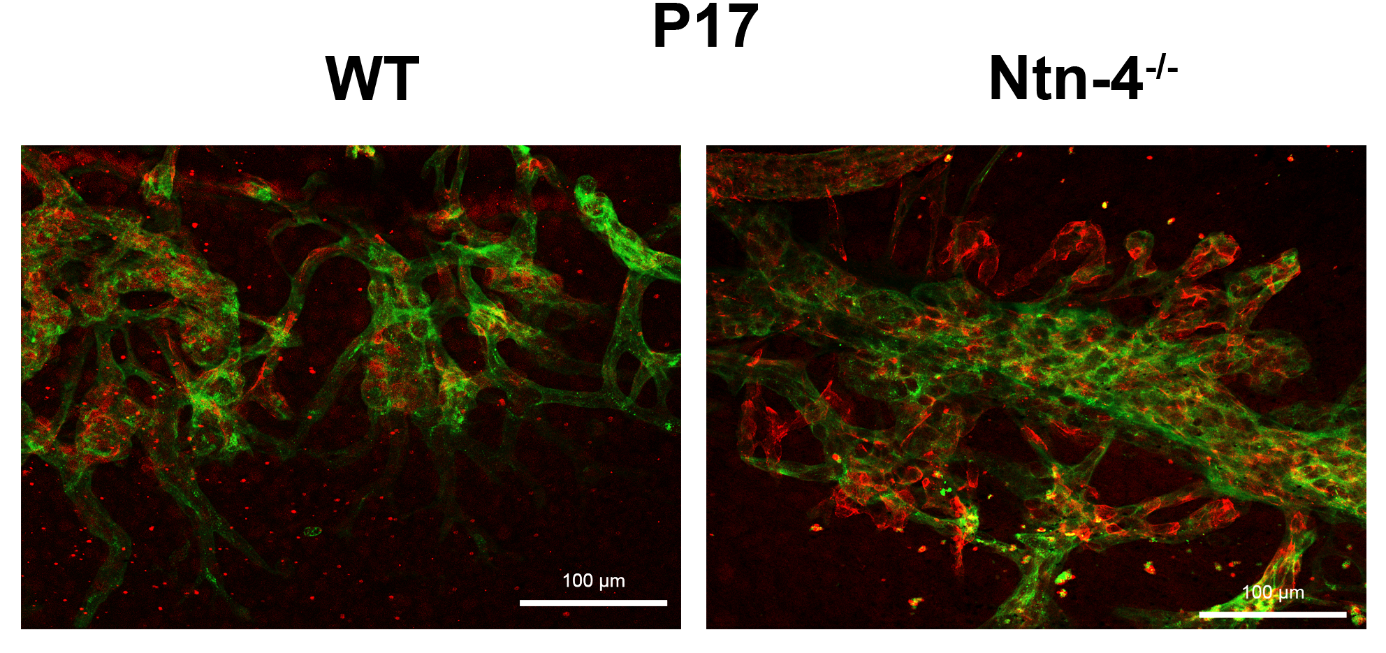


**Suppl. Fig. 4: Pericyte distribution in neovascular tufts**

Whole retina mounts stained against Isolectin-IB_4_ (green) and NG2 (neural/glial antigen 2) (red) in WT and *Ntn-4^-/-^* mice at P17. Pericytes (red) are present on pathological neovascular vessels (green) in both genotypes. Scale bar indicates 100 µm.


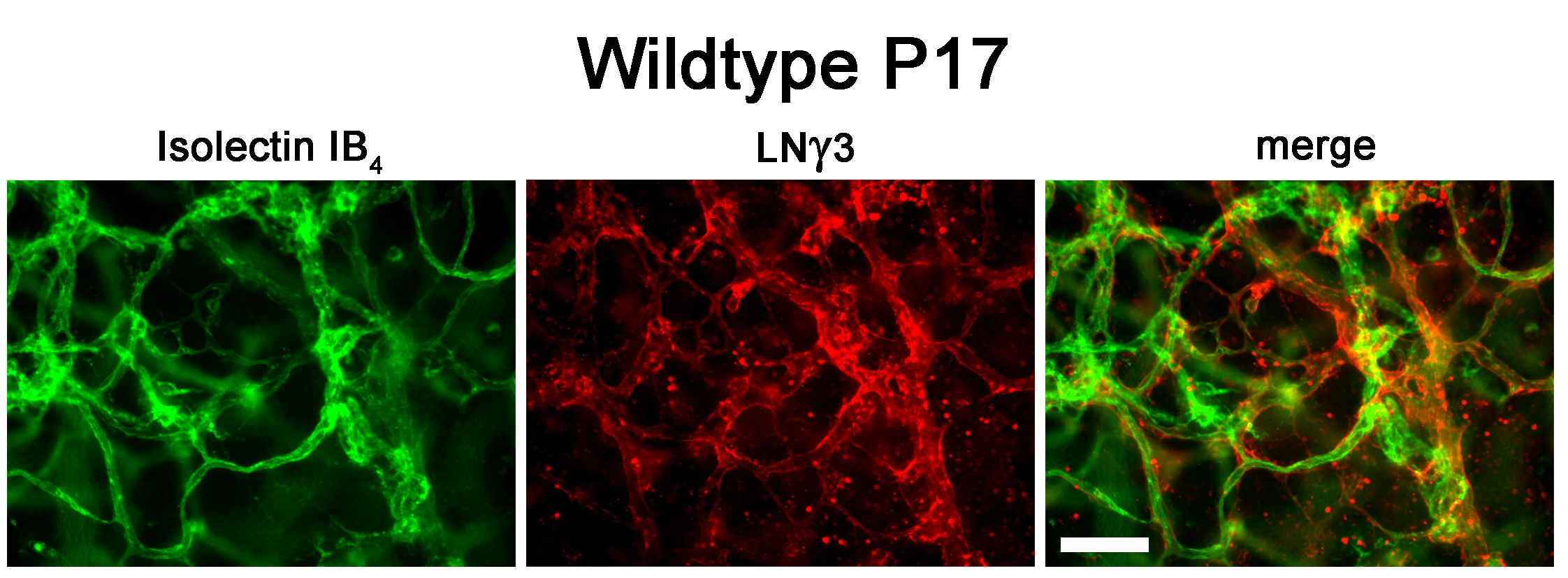


**Suppl. Fig. 5: Partial absence of laminin γ3 in neovascular tufts**

Isolectin-IB_4_ (green) and laminin γ3 (red) co-staining in WT mice at P17. Only a subset of pathological vessels are both stained with Isolectin- IB_4_ and laminin γ3.
Scale bar indicates 25 µm.


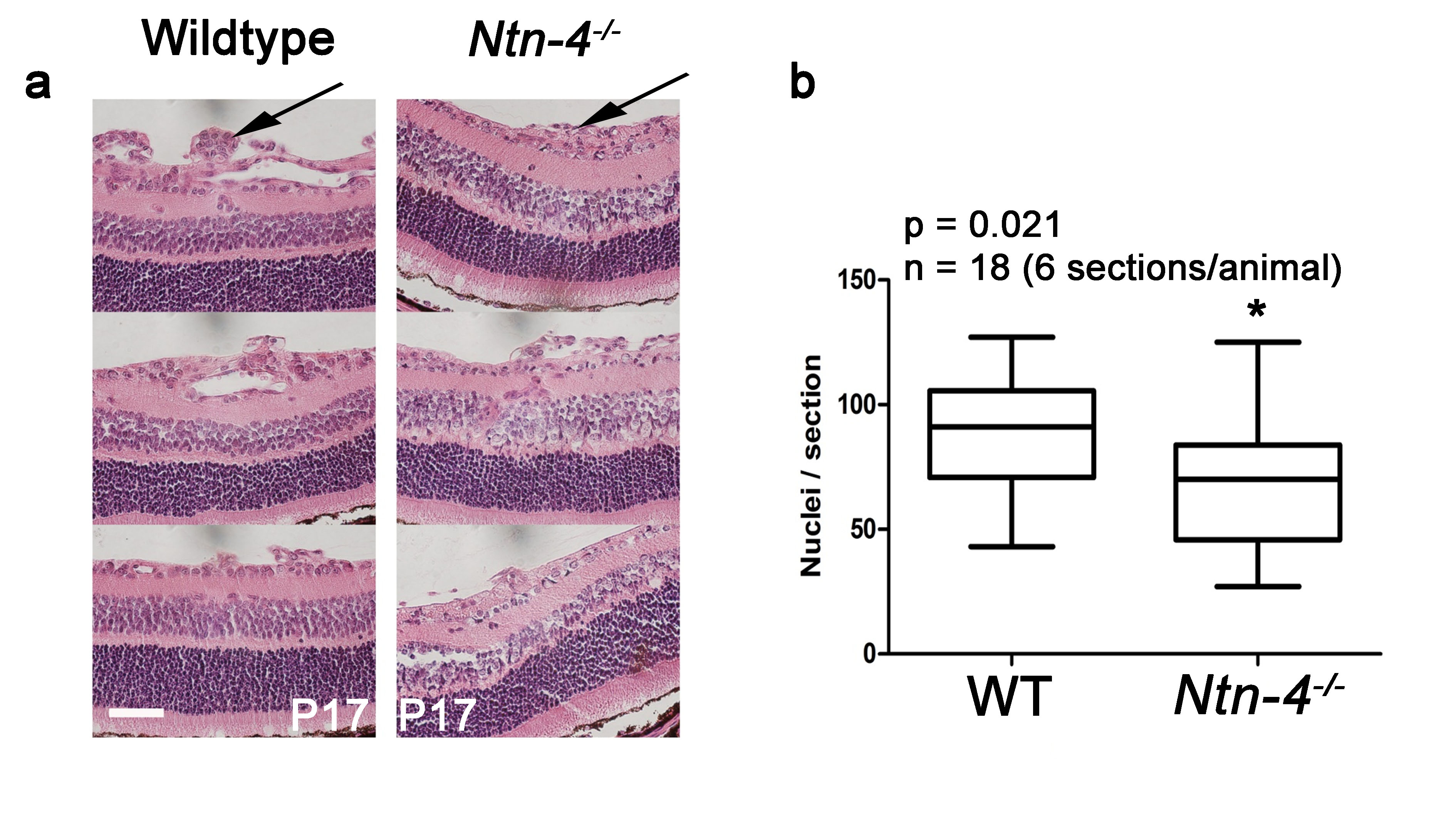


**Suppl. Fig. 6: Paraffin sections through WT and *Ntn-4^-/-^* retinas**

1. HE-stained paraffin section examples of WT mice (left panel) and *Ntn-4^-/-^* mice (right panel) showing vascular tufts penetrating the inner limiting membrane (arrows).
2. Quantification of pre-retinal nuclei of the neovascular tufts. There are less pre-retinal nuclei in *Ntn 4^-/-^* mice.

N is the number of sections counted and came from three mice, each.


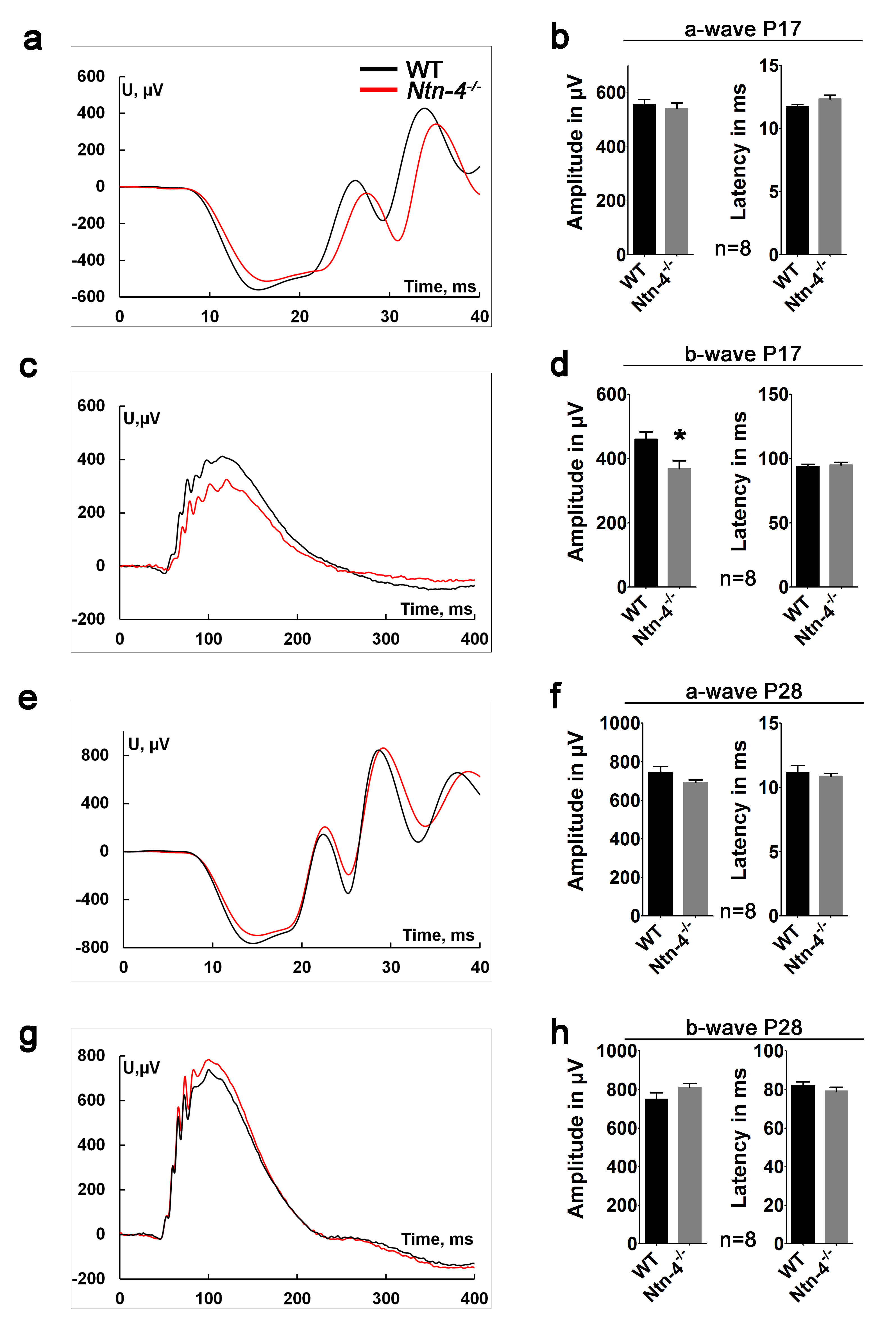


**Suppl. Fig. 7: Ganzfeld-ERGs at P17 and P28 in WT and Ntn-4^-/-^ mice at normoxia**

With the exception of the b-wave amplitude at P17 there is no difference in the ERG response due to different genotypes. Contrary to the increased, but not significant b-wave amplitude in *Ntn-4^-/-^* mice after OIR there is a statistically significant decrease in *Ntn-4^-/-^* mice at normoxia when compared to the WT.
